# Defining the impact of exoribonucleases in the shift between exponential and stationary phases

**DOI:** 10.1038/s41598-019-52453-6

**Published:** 2019-11-07

**Authors:** Vânia Pobre, Susana Barahona, Tatiane Dobrzanski, Maria Berenice Reynaud Steffens, Cecília M. Arraiano

**Affiliations:** 1 Instituto de Tecnologia Química e Biológica António Xavier, Universidade Nova de Lisboa, Av. da República, 2780-157 Oeiras, Portugal; 2 Department of Biochemistry and Molecular Biology, Federal University of Paraná (UFPR), Curitiba, PR Brazil

**Keywords:** Gene expression, Microbial genetics

## Abstract

The transition between exponential and stationary phase is a natural phenomenon for all bacteria and requires a massive readjustment of the bacterial transcriptome. Exoribonucleases are key enzymes in the transition between the two growth phases. PNPase, RNase R and RNase II are the major degradative exoribonucleases in *Escherichia coli*. We analysed the whole transcriptome of exponential and stationary phases from the WT and mutants lacking these exoribonucleases (Δ*pnp*, Δ*rnr*, Δ*rnb*, and Δ*rnb*Δ*rnr*). When comparing the cells from exponential phase with the cells from stationary phase more than 1000 transcripts were differentially expressed, but only 491 core transcripts were common to all strains. There were some differences in the number and transcripts affected depending on the strain, suggesting that exoribonucleases influence the transition between these two growth phases differently. Interestingly, we found that the double mutant RNase II/RNase R is similar to the RNase R single mutant in exponential phase while in stationary phase it seems to be closer to the RNase II single mutant. This is the first global transcriptomic work comparing the roles of exoribonucleases in the transition between exponential and stationary phase.

## Introduction

Bacteria often transition between growing rapidly (exponential phase) to slower growth (stationary phase) depending on the nutrient availability. This transition requires that the cells undergo massive transcriptomic rearrangements. The vast majority of the transcripts that are expressed in exponential phase are repressed in stationary phase and there is the activation of many others in stationary phase^1–3^. The transition between these two phases is usually accompanied by physiological and morphological changes and many transcripts are differentially expressed between the two phases^1^. Moreover, stationary phase is considered a stress condition and many of the transcripts that are highly expressed during this phase are stress related transcripts^2–5^.

Exoribonucleases are key elements in the transition between exponential and stationary phase since they promote the rapid degradation of RNA in the cell. Therefore they promote the reduction of proteins that are not needed in one phase and recycle nucleotides essential for the expression of new RNAs^6,7^. In *E. coli* PNPase, RNase II and RNase R are the major 3′-5′ exoribonucleases. The deletion of one of these exoribonucleases does not affect viability of the cells however the double deletion mutants of PNPase/RNase II and PNPase/RNase R are not viable^8,9^. In contrast, the double mutant RNase II/RNase R is viable but has not yet been analyzsed. The roles of these exoribonucleases have been studied in both growth phases^10,11^ however most studies are focused in specific RNA targets and mechanisms.

RNase R and RNase II belong to the same family of enzymes and therefore have some functional and structural similarities^12,13^. Both are hydrolytic enzymes. However, only RNase R is able to degrade structured RNA, mainly due to its helicase activity^14,15^, while in RNase II the double-stranded RNA cannot access the catalytic pocket^13^. This inability of RNase II to degrade secondary structures^16^ can lead to the protection of certain structured RNAs; RNase II is very effective in the removal of poly(A) tails and thus impairs the action of the other exoribonucleases^17–20^. Both RNase II and RNase R proteins can be acetylated although this is growth specific for both enzymes^21,22^. In exponential phase RNase II and RNase R were found to negatively affect cell motility and their absence leads to an increase in the cells ability to produce biofilms^23^. RNase R interacts with bacterial ribosomes^24^ and in exponential phase the vast majority of RNase R proteins are bound to the ribosomes^25^. PNPase belongs to the PDX family of enzymes which have phosphorolytic activity, but PNPase can also act as a polymerase and synthesize long heteropolymeric tails if the concentration of inorganic phosphate is very low^26–28^. PNPase can form multiprotein complexes such as the degradosome, an extremely efficient degradative machine^29–32^. Moreover, PNPase is the major exoribonuclease responsible for the degradation of small RNAs that are not bound to their targets or the RNA chaperone Hfq^33–36^. On the other hand, PNPase can also protect sRNAs from degradation by establishing complexes with the sRNA and the Hfq protein^37^.

In this work we used a whole transcriptome approach to determine the differences between the exponential and stationary phases of *E. coli* WT cells and the exoribonuclease mutants (Δ*rnb*, Δ*rnr*, Δ*pnp* and Δ*rnb*Δ*rnr*). This study is the first comparative transcriptomic analysis of these two growth phases. Moreover, it is the first global transcriptomic analysis of the double mutant RNase II/RNase R. Surprisingly we found that the double mutant Δ*rnb*Δ*rnr* is not only a merger of the single mutations, but it appears that in exponential phase the double mutant is more similar to the single mutant Δ*rnr* while in stationary phase the double mutant is more similar to Δ*rnb* single mutant. Moreover, we found that independently of the strain analysed there are more than 1000 transcripts significantly differentially expressed between the exponential and stationary phases. Of all these transcripts there are 491 core transcripts that are affected in all strains but unexpectedly there are transcripts that are only affected in the exoribonuclease mutants. Furthermore, when analysing the biological pathways affected between the exponential and stationary phases for all strains, we found that there are processes that are specific for each of the exoribonuclease mutants.

## Results

### Analysis of the RNase II/RNase R double mutant in exponential and stationary phase

**Figure 1:**
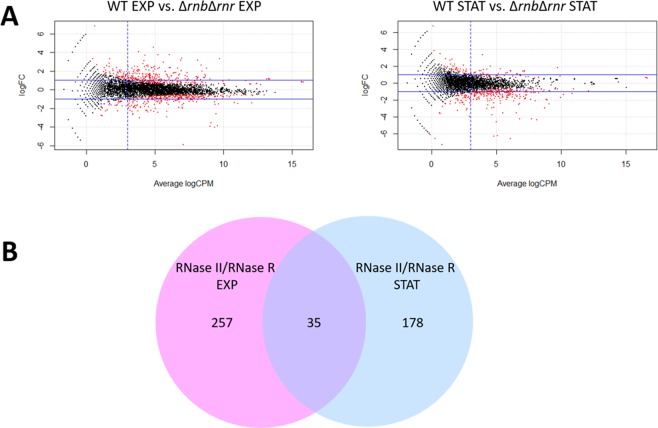
RNase II/RNase R double mutant in exponential and stationary phase. (**A**) MA scatterplot comparing wild-type (WT) with Δ*rnb*Δ*rnr* mutant in exponential and stationary phase. LogFC is the log2 of the fold change for each transcript, average LogCPM is the relative expression value for each transcript. Transcripts in red were considered significantly differentially expressed (FDR < 0.05), the two horizontal blue lines correspond to a fold-change of 2 and the vertical blue line correspond to the LogCPM of 3. These lines represent the filtration steps done to obtain the final list of differentially expressed transcripts. (**B**) Venn diagram comparing the number of transcripts that are significantly differentially expressed between the WT and the double mutant in exponential and stationary phases.

The global evaluation of the absence of hydrolytic RNA degradation activity has never been performed. Therefore, in this work we analysed the impact of the deletion of both RNase II and RNase R in both exponential and stationary phases of growth. We sequenced the total RNA (RNA-Seq) extracted from wild-type (WT) cells and the double mutant Δ*rnb*Δ*rnr* (RNase II/RNase R) in both exponential and stationary phases. We then did a global transcriptomics analysis to determine the transcripts that were affected by the deletion of these two enzymes. The fold-change for each transcript between the WT and the double mutant was calculated and plotted in a MA scatterplot (Fig. 1A). When analysing the RNA-Seq data it is clear that many of the transcripts that are significantly differentially expressed between the different mutants and the WT do not have a high expression value and do not present a high fold-change, for this reason we filtered our results to obtain a list of highly expressed transcripts (LogCPM > 3) and with a fold-change higher than two between the mutant and the WT. We found a total of 292 (Table S1) and 191 (Table S2) differentially expressed transcripts in exponential and stationary phase, respectively. In exponential phase the number of transcripts that are up-regulated (61.3%) is higher than the number of down-regulated (38.7%) transcripts for the double mutant. On the other hand, in stationary phase most of the transcripts are down-regulated (94.8%) with only a very small percentage (5.2%) being up-regulated (Table 1). We have previously observed this discrepancy in up-regulated and down-regulated transcripts in the Δ*rnb* mutant in exponential phase^23^. To be able to established a better comparison of our current data with the data previously published^23^ we re-analysed the RNA-Seq data with the same workflow used in this study (see Methods section). The number of total differentially expressed transcripts that have high expression values (LogCPM > 3) and a fold-change higher than 2 between the WT and the different exoribonuclease mutants, as well as the percentage of up and down-regulated transcripts are summarized in Table 1. Although we obtained slightly different number of transcripts differentially expressed when comparing the WT with the different exoribonuclease mutants in exponential phase, overall the data followed the same tendency and the functional annotation was also identical with the previously published data.

**Table 1:** Number of filtered differentially expressed transcripts between the WT and the exoribonucleases mutants in exponential and stationary phase.

Exoribonuclease mutant	Exponential phase			Stationary phase		
	Total	Up (%)	Down (%)	Total	Up (%)	Down (%)
Δ*rnb*	113	18.6	81.4	178	11.8	88.4
Δ*rnr*	440	63.0	37.0	74	16.2	83.8
Δ*pnp*	630	48.4	51.6	454	35.2	64.8
Δ*rnb*Δ*rnr*	292	61.3	38.7	191	5.2	94.8

Therefore, if we compare the number of up-regulated and down-regulated transcripts of the double mutant with the single mutants we can see that in exponential phase the double mutant is very similar to the Δ*rnr* single mutant (Table 1). However, in stationary phase the double mutant is closer to the Δ*rnb* single mutant although in this growth phase the three mutants are quite similar with the number of down-regulated transcripts being much higher than the number of up-regulated transcripts (Table 1, Tables S3 and S4, Fig. S1A,B). This seems to indicate that the double mutant Δ*rnb*Δ*rnr* is more similar to the single mutant Δ*rnr* in exponential phase but in stationary phase the double mutant might be closer to Δ*rnb* single mutant. The Δ*pnp* mutant does not present such high differences with regards to the number of up and down-regulated transcripts in neither growth phase, although it is the mutant that presents the higher number of total differential expressed transcripts in both growth phases (Table 1, Table S5, Fig. S1C).

We then compared the transcripts that are being affected by the deletion of both exoribonucleases in exponential and stationary phase. We found that only 35 transcripts are common to both growth phases while most of the transcripts that are significantly affected by the deletion of both RNase II and RNase R are growth phase specific (Fig. 1B). These results seem to suggest that the double mutant is not simply a merger of the single mutants and there must be compensation mechanisms that are dependent of the growth phase.

### Comparison of the phosphorolytic and hydrolytic activities of the exoribonucleases

**Figure 2:**
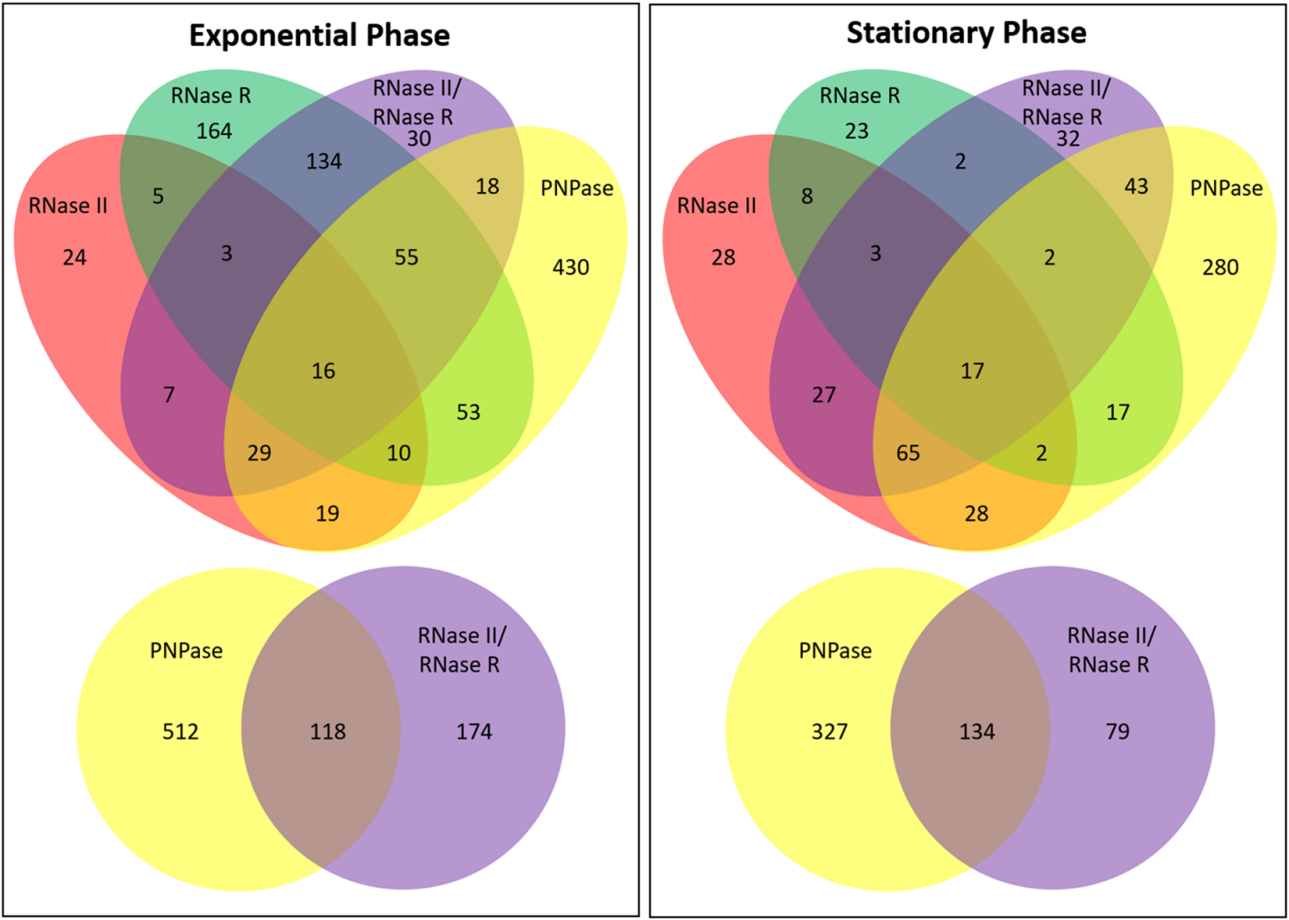
Comparison of the phosphorolytic and hydrolytic activities of the exoribonucleases. Venn diagram comparing the number of transcripts that are significantly differentially expressed between the WT and the different exoribonuclease mutants in exponential and stationary phases.

RNase II and RNase R belong to the same family of enzymes and although they have distinct modes of action, they are both hydrolytic enzymes. On the other hand, PNPase is a phosphorolytic enzyme and belongs to a different family of enzymes. Since there is some overlap between the three enzymes we decided to analyse if this overlay was influenced by the activity differences (hydrolytic *vs* phosphorolytic) between the three exoribonucleases on both exponential and stationary phase. For this we compared the filtered lists of differentially expressed transcripts between the WT and the different mutants to identify which transcripts were being affected in two or more mutants. PNPase deletion mutant presented the higher number of affected transcripts in both growth phases suggesting that deletion of the phosphorolytic exoribonuclease has a more significant impact than even the deletion of both degradative hydrolytic exoribonucleases (Table 1). In fact, the double mutant Δ*rnb*Δ*rnr* seems to affect much less transcripts than the sum of transcripts that change in single mutants in both growth phases. When comparing the transcripts affected only a small number of transcripts are affected in all exoribonuclease mutants (Fig. 2). In exponential phase only 16 transcripts are affected in all mutants while in stationary phase only 17 transcripts were common to all mutants. However, in exponential phase the double mutant and the RNase R single mutant have 134 transcripts in common while in stationary phase the number of common transcripts between the double mutant and RNase R single mutant is much less significant (only 2 transcripts). On the other hand, the number of common transcripts between the double mutant and the RNase II mutant is higher in stationary phase (27 transcripts) than in exponential phase (7 transcripts). These results further demonstrate that the double mutant is closer to RNase R single mutant in exponential phase but in stationary phase the double mutant seems to be closer to RNase II single mutant. Furthermore, when comparing the double mutant to the PNPase single mutant we found that there is a significant number of transcripts that are affected in both mutants. However, this is more relevant for the double mutant with 118 transcripts of a total of 292 transcripts (40%) that are affected by the deletion of the hydrolytic exoribonucleases also being affected by PNPase deletion in exponential phase, while in stationary phase this is even more significant with 134 transcripts of a total of 213 transcripts (63%) also being affected in the PNPase mutant (Fig. 2). On the other hand, PNPase deletion seems to affect many transcripts that are not affected when both RNase II and RNase R are deleted. Overall this result suggests that there is a definitive overlap between the actions of the hydrolytic and the phosphorolytic exoribonucleases and that the phosphorolytic activity seems to have a broader role over the hydrolytic activity in the cell metabolism, especially in stationary phase.

### Global changes between exponential and stationary phase

**Figure 3:**
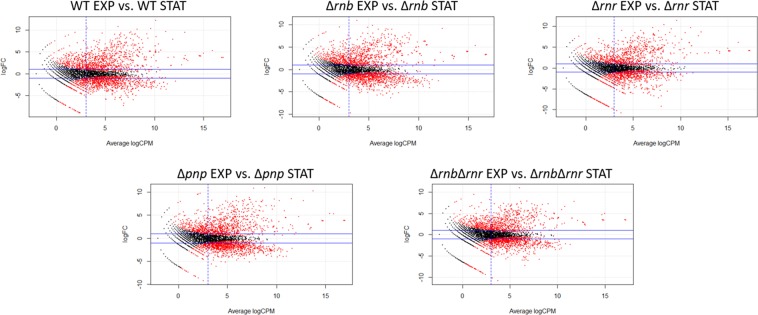
Global changes between exponential and stationary phase. MA scatterplots comparing the exponential with stationary phase for each of the strains. LogFC is the log2 of the fold change for each transcript, average LogCPM is the relative expression value for each transcript. Transcripts in red were considered significantly differentially expressed (FDR < 0.05), the two horizontal blue lines correspond to a fold-change of 2 and the vertical blue line correspond to the LogCPM of 3. These lines represent the filtration steps done to obtain the final list of differentially expressed transcripts.

The bacteria life cycle often involves a transition from exponential (rapid growth) to stationary phase (slow growth). This transition from one growth phase to the other requires a massive adjustment of the bacteria transcriptome^1^ that translates into a reorganization of the bacteria physiology and morphology^4^. In this work we analysed the full transcriptomic changes between the exponential and stationary phases in the WT and the different exoribonuclease mutants. As expected, we found a large number of transcripts that are being differentially expressed when comparing the same strain in the exponential *vs*. the stationary phase in all strains analysed (Fig. 3). Comparing the different MA scatterplots, there is a similar pattern of transcripts distribution, however in the double mutant it is possible to observe that the distribution of the up-regulated transcripts is slightly reduced than in the other strains. We again filtered the differentially expressed transcripts and found that in all strains there is around 1000 transcripts being affected when comparing the exponential and the stationary phase (Tables 2 and S6–S10). Moreover, we found that most of these transcripts are down-regulated in stationary phase while a smaller number are up-regulated (Tables 2 and S6–S10). The difference in distribution pattern for the double mutant does however translate to a lower number of up-regulated transcripts (173 transcripts) between exponential and stationary phase while all other strains have more than 200 transcripts being up-regulated with the WT having more than 350 transcripts up-regulated (Table 2). It is also noteworthy the fact that are several highly expressed transcripts with very high fold-changes (Fig. 3, Tables S6–S10) because when transitioning from exponential to stationary phase bacteria need to significantly decrease their cellular metabolism. These results are in agreement with other reports^1,4^ and confirm how drastic the changes from exponential to stationary phase are. A growth curve for all strains showed that there are differences in the growth of the wild-type cells and the different mutants and as expected the double mutant did present the slower growth rate of all strains (Fig. S3).

**Table 2:** Number of filtered differentially expressed transcripts between exponential and stationary phase in the WT and the exoribonucleases mutants.

Exoribonuclease mutant	Up-regulated	Down-regulated	Total
WT	357	813	1170
Δ*rnb*	288	896	1184
Δ*rnr*	249	780	1029
Δ*pnp*	261	950	1211
Δ*rnb*Δ*rnr*	173	901	1074

### Overlap between the exponential and stationary phases in the WT and exoribonuclease mutants

**Figure 4:**
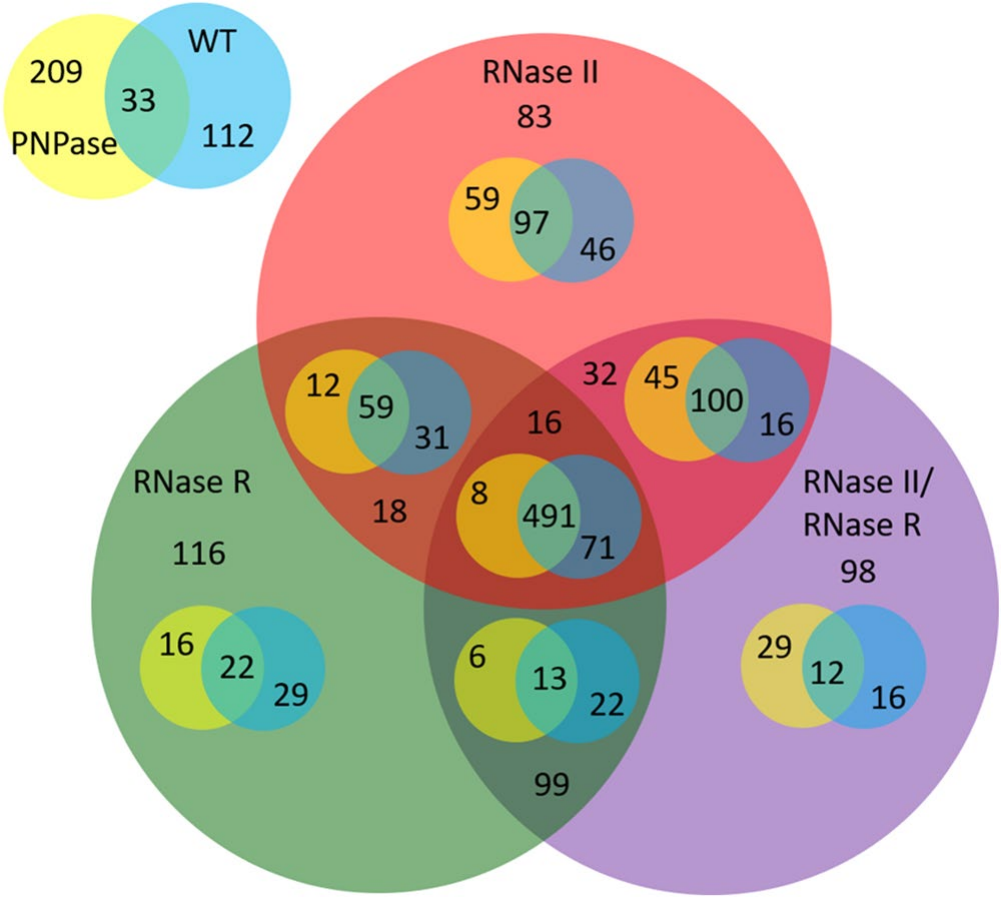
Overlap between the exponential and stationary phases in the WT and exoribonuclease mutants. Venn diagram comparing the number of transcripts that are significantly differentially expressed between the exponential and the stationary phase for the WT and the different exoribonuclease.

The exoribonucleases have different roles during the exponential and the stationary phase of growth. For example, during exponential phase PNPase protects some small RNAs while during stationary phase it was found that PNPase would degrade those small RNAs^33,38^. We then compared the transcripts that were being affected between the two growth phases in the different strains. We found that 491 transcripts are affected independently of the strain (Fig. 4), suggesting that these core transcripts are essential for the adaptation of the bacteria to the different growth conditions. It was however surprising the fact that in the PNPase mutant there are 209 transcripts that do not overlap with any other strain. This number is significantly higher than the number of transcripts in the other strains that do not overlap (WT – 112, RNase II – 83, RNase R – 116 and RNase II/RNase R – 98). It is also noteworthy that from the transcripts that do overlap there is only 8 transcripts that overlap with all exoribonucleases and not with the WT (Fig. 4) once again suggesting that there are compensatory mechanisms when the exoribonucleases are not present.

### Biological processes affected when cells transition between exponential and stationary phase

**Figure 5:**
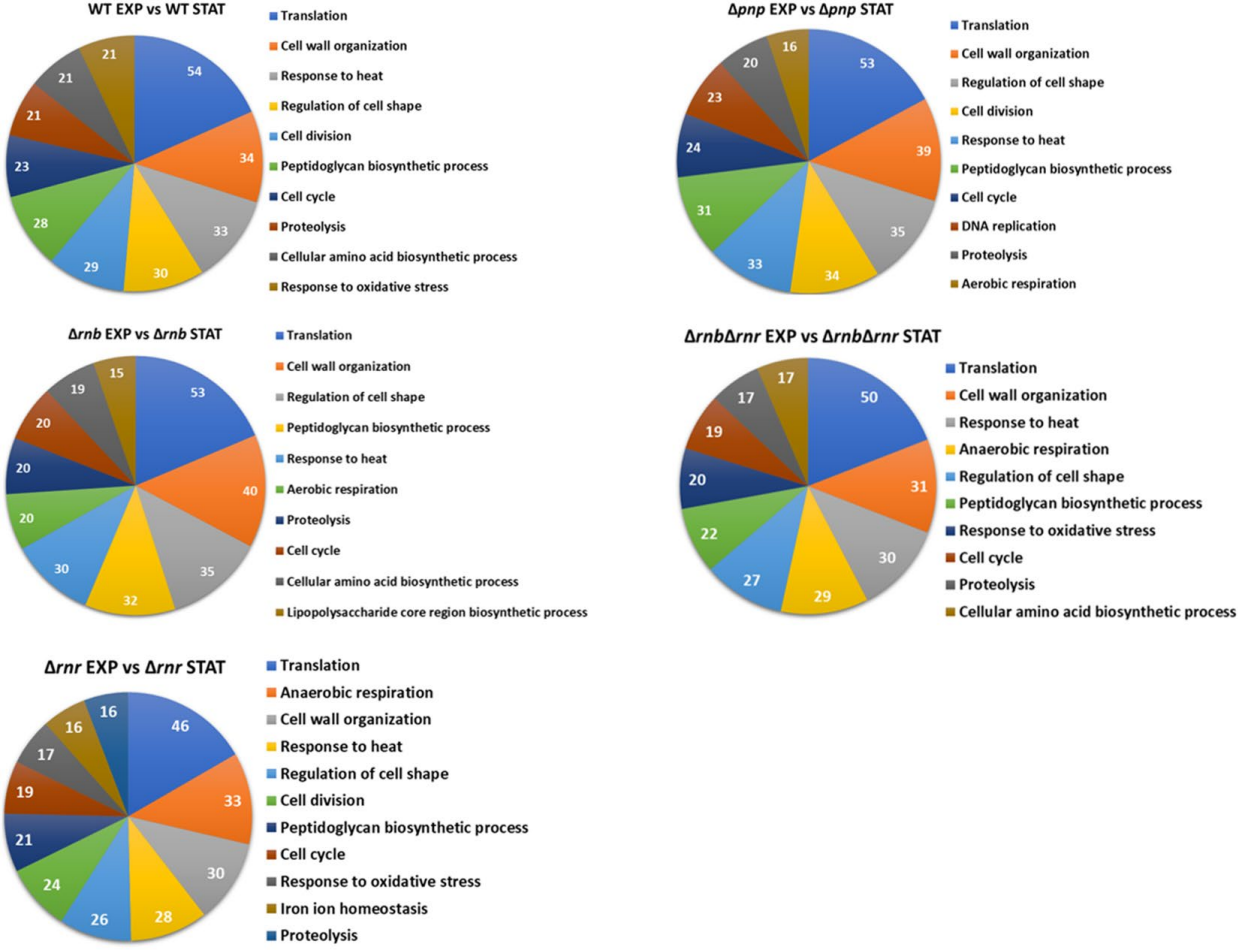
Biological processes affected when the WT and the exoribonuclease mutants enter in stationary phase. Functional annotation of the differentially expressed transcripts between exponential and stationary phase for each of the strains. Transcripts were grouped into different functional categories but only the Gene Ontology category of biological process is represented.

**Figure 6:**
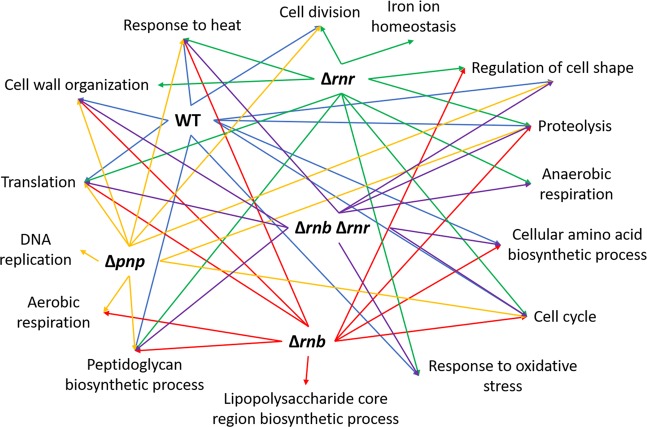
Biological processes affected in the transition between exponential and stationary phases. Diagram representing the connection between the different biological processes affected when comparing the exponential and the stationary phases. We compared the WT in the two phases and showed the relevant processes which were affected; these comparisons were repeated in each mutant strain. Each strain is represented by a different colour.

Bacteria have a unique capability to rapidly adjust its transcriptome to different growth conditions and as mentioned before more than 1000 transcripts are being affected when comparing the transcriptome of cells in exponential and stationary phase. We then decided to analyse exactly what were these transcripts and their roles in the cell. For this we did a functional annotation analysis of all the transcripts affected in the different strains. Previous work had already shown that in exponential phase the exoribonucleases affected processes related to motility and biofilm formation^23^. We also analysed the processes affected by the exoribonucleases in stationary phase when compared to the WT and found that in this growth phase the TCA cycle is affected in the RNase II, PNPase and the double mutant but not in the RNase R mutant (Fig. S2). Moreover, we found that all exoribonuclease mutants affected transcripts related to stress response mechanisms. These results suggest that in stationary phase the exoribonucleases have a greater role in the cell response to stress. Next, we analysed the biological processes that were being affected between the exponential and stationary phase for all strains (Fig. 5). As expected in all strains translation was found to be the main process affected. This process is essential for the cells to be able to transition from one growth phase to the other since the cell needs to control the amount of proteins being produced to be able to survive. Furthermore, we found that all strains had most of the same processes being affected. To better analyse this result, we constructed a diagram with the distribution of the different processes affected in the different strains (Fig. 6). Interestingly we found that there were some unique processes being affected in specific strains. Iron ion homeostasis was found to be affected only in the RNase R mutant, the biosynthesis of lipopolysaccharides was affected only in the RNase II mutant and DNA replication was found to be affected only in the PNPase mutant. Noteworthy the double mutant (RNase II/RNase R) does not appear to affect neither the iron ion homeostasis nor the biosynthesis of lipopolysaccharides suggesting that in the double mutant there must exist compensatory mechanisms that are not activated in the single mutants. Another interesting result is the aerobic and anaerobic respiration processes that are not affected when comparing the WT cells in exponential and stationary phase but are affected by two of the exoribonuclease mutants (Fig. 6). These results show that in the exoribonuclease mutants there are processes affected between the exponential and stationary phase which are not affected in the WT cells. Moreover, and in agreement with our previous results we can also found that there are biological processes that are affected by the double mutant and only by RNase II or RNase R. For example, RNase R and the double mutant affect the response to oxidative stress that is not affected by the RNase II mutant, while the cellular amino acid biosynthetic process is affected by the double mutant and the RNase II mutant but not by the RNase R mutant. To validate some of these results we did qPCR of 27 different transcripts that were significantly affected and that belong to different functional categories. We found a good correlation between the qPCR results and the RNA-Seq data for most of the transcripts analysed (Table 3). However, when comparing the exponential phase with the stationary phases some transcripts had extremely low expression in stationary phase and therefore is not possible to validate by qPCR.

**Table 3:** Comparison between the values for fold change of some genes using RNA-Seq and qPCR.

Comparison	Functional categories	Transcript	Mutant	RNA-Seq^a^	qPCR^a^
WT vs. exoribonuclease mutant	Stress response transcripts	*acnA*	Δ*rnb*	0.39	0.49
			Δ*rnr*	0.75	0.80
			Δ*pnp*	0.52	0.37
			Δ*rnb*Δ*rnr*	0.27	0.66
		*clpB*	Δ*rnb*	0.36	0.45
			Δ*rnr*	0.50	0.73
			Δ*pnp*	0.44	0.51
			Δ*rnb*Δ*rnr*	0.42	0.86
		*dnaK*	Δ*rnb*	0.24	0.49
			Δ*rnr*	0.45	0.83
			Δ*pnp*	0.64	0.78
			Δ*rnb*Δ*rnr*	0.29	0.54
		*groL*	Δ*rnb*	0.27	0.39
			Δ*rnr*	0.51	0.68
			Δ*pnp*	0.54	0.60
			Δ*rnb*Δ*rnr*	0.27	0.55
		*htpG*	Δ*rnb*	0.33	0.45
			Δ*rnr*	0.52	0.74
			Δ*pnp*	0.35	0.55
			Δ*rnb*Δ*rnr*	0.35	0.59
		*ibpA*	Δ*rnb*	0.33	3.67
			Δ*rnr*	0.42	0.49
			Δ*pnp*	0.57	0.58
			Δ*rnb*Δ*rnr*	0.41	4.12
		*icD*	Δ*rnb*	0.28	0.49
			Δ*rnr*	0.33	0.58
			Δ*pnp*	0.27	0.20
			Δ*rnb*Δ*rnr*	0.19	1.05
		*katE*	Δ*rnb*	0.33	0.35
			Δ*rnr*	0.55	1.17
			Δ*pnp*	0.22	0.07
			Δ*rnb*Δ*rnr*	0.25	0.45
		*katG*	Δ*rnb*	0.29	0.41
			Δ*rnr*	0.77	0.42
			Δ*pnp*	0.21	0.10
			Δ*rnb*Δ*rnr*	0.23	0.71
	TCA cycle transcripts	*aceA*	Δ*rnb*	0.19	0.26
			Δ*rnr*	0.71	0.63
			Δ*pnp*	0.20	0.83
			Δ*rnb*Δ*rnr*	0.13	0.13
		*sucD*	Δ*rnb*	0.36	0.31
			Δ*rnr*	0.73	0.60
			Δ*pnp*	0.33	0.94
			Δ*rnb*Δ*rnr*	0.36	0.32
		*sdhC*	Δ*rnb*	0.30	0.56
			Δ*rnr*	0.24	0.23
			Δ*pnp*	0.34	1.34
			Δ*rnb*Δ*rnr*	0.36	0.34
		*gltA*	Δ*rnb*	0.28	0.26
			Δ*rnr*	0.36	0.33
			Δ*pnp*	0.22	0.86
			Δ*rnb*Δ*rnr*	0.29	0.19
Exponential phase vs. Stationary phase	Iron ion homeostasis	*fhuB*	Δ*rnr*	0.035	0.011
		*fecC*	Δ*rnr*	0.029	0.006
		*fre*	Δ*rnr*	0.046	0.002
	Anaerobic respiration	*frdA*	Δ*rnr*	0.026	0.004
		*napA*	Δ*rnr*	0.005	0.002
		*dmsA*	Δ*rnr*	0.009	0.005
		*dmsA*	Δ*rnb*Δ*rnr*	0.006	0.008
	DNA replication	*dnaB*	Δ*pnp*	0.037	0.008
		*mukB*	Δ*pnp*	0.045	0.009
		*holA*	Δ*pnp*	0.062	0.050
	LPS biosynthesis	*waaC*	Δ*rnb*	0.015	0.006
		*waaY*	Δ*rnb*	0.059	0.022
		*lpxK*	Δ*rnb*	0.046	0.009
	Aerobic respiration	*cyoB*	Δ*rnb*	0.014	0.005
		*rsxC*	Δ*rnb*	0.036	0.005

^a^Fold Changes were calculated as the ratio of mutant to WT or the ratio between exponential and stationary phases. Values above 1 correspond to up-regulated transcripts while values below 1 correspond to down-regulated transcripts.

## Discussion

In this work we analysed the role of exoribonucleases in the transition between exponential and stationary phases since these enzymes are key players in RNA degradation mechanisms and therefore important for the rapid transition between these growth phases. The growth curves for all strains is similar however the deletion mutants do require more time to reach stationary phase than the wild-type, moreover, as expected the double mutant presented the slower growth of all strains (Fig. S3). This is in agreement with our findings where we showed that the double mutant RNase II/RNase R affects the expression of many transcripts in both exponential and stationary phases although the number of transcripts significantly differentially expressed between the WT and the double mutant is higher in exponential than in stationary phase (Fig. 1, Table 1). However, the percentage of up-regulated and down-regulated transcripts for the double mutant is quite different when comparing both growth phases. In exponential phase the number of up-regulated and down-regulated transcripts is not so different while in stationary phase the vast majority of the transcripts are down-regulated (Tables 1, S1 and S2). Previous work had already shown that in exponential phase the RNase II mutant had a higher percentage of down-regulated transcripts than up-regulated^23^ and we also found the same tendency for this mutant in stationary phase (Table 1, Fig. S1 and Table S3). However, this striking difference in the number of up-regulated and down-regulated transcripts for the double mutant only happens in stationary phase suggesting that in exponential phase the double mutant is similar to the RNase R single mutant while in stationary phase the double mutant seems to be closer to the RNase II single mutant. This is further supported by the fact that the pathways affected in the double mutant in stationary phase are almost identical to the RNase II single mutant (Fig. S2) and when comparing the common transcripts between the RNase II, RNase R and the RNase II/RNase R mutants in both exponential and stationary phases (Fig. 2). This duality of the double mutant might be related to the fact that both RNase II and RNase R are affected in different ways in both growth phases. In exponential phase RNase R is acetylated^21^ and is bound to the ribosomes^24,25^ while in stationary phase RNase R is no longer acetylated nor is bound to the ribosomes^25^. RNase II is acetylated both in exponential and stationary phase however the levels of acetylation are much higher in stationary phase^22^. The acetylation of these enzymes greatly affects their activity and availability to degrade RNA influencing their roles in both growth phases and this might explain the differences observed for the double mutant in both exponential and stationary phases.

Interestingly when comparing the phosphorolytic (Δ*pnp*) and hydrolytic (Δ*rnb*Δ*rnr*) activities of the exoribonucleases we found that PNPase affects many more transcripts than the double mutant in both exponential and stationary phase (Fig. 2 and Table 1). Although there is some overlap between these two mutants the vast majority of the transcripts affected in the PNPase mutant do not overlap with any other exoribonuclease mutant. This might be due to the role of PNPase in the metabolism of the small RNAs. It is known that PNPase affects sRNAs in both exponential^37,39^ and stationary phases^33,34,36,39^ and in fact in stationary phase PNPase was found to be the major enzyme responsible for the degradation of free sRNAs^33,34^. Since RNase II and RNase R do not seem to have any major role in the metabolism of sRNAs^35^ it is likely that the higher number of transcripts being affected by the absence of PNPase is due to its role in these specific class of RNAs.

Both exponential and stationary phases have been extensively studied however this is the first work where a direct comparison of the transcriptome for the two growth phases is conducted. Our work showed that there are massive transcriptomic differences between the two growth phases, independently of the strain analysed. Overall, we found that more than 1000 transcripts are differentially expressed when comparing the exponential to the stationary phase (Table 2) a result that is in agreement with a previous study^1^. Moreover, the number of transcripts that are down-regulated in stationary phase is much higher than the number of transcripts that are up-regulated. This is to be expected since in stationary phase the nutrient availability is very low and therefore bacteria needs to shut down most of its transcripts in order to survive^1–3^. On the other hand, since stationary phase is a stressful condition bacteria needs to express transcripts that can help them to survive^4,5^. We found that there is a core of 491 transcripts that are always affected when comparing the exponential and stationary phases for all strains (Fig. 4). We can then conclude that these transcripts are of the upmost importance for the cell transition between the two growth phases. Interestingly we found that the PNPase mutant has a much higher number of unique significantly differentially expressed transcripts between exponential and stationary phase than any other strain (Fig. 4). This might suggest that PNPase itself can have a role in the transition between the exponential and stationary phase. However, this might be due to the different role that PNPase has in the sRNAs in exponential and stationary phase. In fact, PNPase was found to protect some sRNAs in exponential phase while in stationary phase it would degrade those same sRNAs^33,37^. This duality of roles can account for the high number of unique transcripts found for the PNPase mutant.

A functional analysis of the transcripts differentially expressed between the exponential and stationary phases reveals that, as expected, this transition affects transcripts related to translation, morphology, and biosynthesis of several compounds (Figs 5 and 6). Surprisingly, we also found that there are specific biological processes being affected only in the exoribonuclease single mutants. DNA replication pathway was found to be affected only in the PNPase mutant and this might be explained by the fact that PNPase is able to degrade ssDNA and was found to be involved in DNA repair mechanisms^40^; moreover PNPase was suggested to participate in the synthesizes of CDP and therefore being directly involved in DNA replication^41^. Iron ion homeostasis was found to be affected when comparing the exponential with the stationary phase only in the absence of RNase R. Although so far no correlation has been described between RNase R and iron metabolism, RNase R is considered a stress protein with its expression being increased in several stress conditions^42^ and therefore it might be possible that RNase R is involved in iron homeostasis. Furthermore, it is noteworthy that *nsrR* gene is co-transcribed with the *rnr* gene and that NsrR is a NO-sensitive repressor from the Rrf2 family that contains an [Fe–S] cluster^43^ suggesting that might exist a co-relation between RNase R and iron that is so far unknown. Although lipopolysaccharide (LPS) biosynthesis was only found to be affected between exponential and stationary phases in the absence of RNase II, this is due to the p-value cut-off applied to the results. In fact, if we allow for a higher p-value this biological process will also appear for all other strains. It is however interesting to see that in the absence of RNase II this process appears but not the cell division suggesting that RNase II might have a role in LPS biosynthesis unknown so far. Besides these unique processes the aerobic and anaerobic processes were also found to be affected between the exponential and stationary phase but only for some strains. The aerobic respiration was affected in the PNPase and RNase II mutants and the anaerobic respiration was affected in the RNase R and double mutant (Figs 5 and 6). PNPase is known to be involved in cellular respiration mechanisms by maintaining mitochondrial homeostasis in mammals^44^, moreover in bacteria PNPase activity is regulated by ATP^45^ linking PNPase to aerobic respiration processes. As for RNase II there is still no known link between this enzyme and aerobic respiration. On the other hand, RNase R has already been implicated in the anaerobic metabolism^46^ and it is likely that the double mutant is also involved in the anaerobic metabolism.

Overall with this work we were able to obtain a broader view of the transcriptomic changes that occur when bacteria transits from the exponential to the stationary phase. Additionally, this work was extremely useful to expand our knowledge of the roles of exoribonucleases in the different growth phases, while at the same time raised many other questions that require further investigation.

## Methods

### Strains and growth conditions

In this work we used *E. coli* K-12 strain MG1693 and its isogenic strains (Table S11) grown at 37 °C, 200 rpm in Luria-Bertani (LB) medium supplemented with thymine (50 μg ml^−1^). Antibiotics were added to the deletion strains as following: kanamycin, 50 μg ml^−1^ (Δ*rnr* and Δ*rnb* Δ*rnr*); tetracycline, 20 μg ml^−1^ (Δ*rnb* and Δ*rnb* Δ*rnr*); streptomycin/spectinomycin 20 μg ml^−1^ (Δ*pnp*). A growth curve for all strains is shown in Fig. S3.

### Total RNA extraction

Overnight cultures were diluted in fresh LB medium to an initial OD600 ~ 0.03 and grown to exponential phase (OD600 ~ 0.5) and to stationary phase (16 h growth). RNA was isolated following the phenol:chloroform extraction protocol as previously described^33^. After the precipitation step in ethanol and 300 mM sodium acetate, RNA was resuspended in MilliQ-water. The integrity of RNA samples was verified with an agarose gel electrophoresis. When necessary to remove contaminant DNA the turbo DNase (Ambion) was used followed by another phenol:chloroform purification step.

### RNA-Seq and data analysis

Total RNA samples (20 μg) were sequenced at Vertis Biotechnologie AG, Germany, with an Illumina HiSeq platform (single end, 50-bp read length, 10 M reads). Vertis Biotechnologie AG depleted the ribosomal RNA molecules using the MICROBExpress Bacterial mRNA Enrichment Kit (Ambion). The RNAs were then fragmented with RNase III and the 5′PPP structures were removed using RNA 5′ Polyphosphatase (Epicentre). Next, an RNA adapter was ligated to the 5′-phosphate of the RNA and the first-strand cDNA synthesis was performed using an oligo(dT)-adapter primer and M-MLV reverse transcriptase. The resulting cDNA was PCR-amplified to about 30 ng/μl using a high-fidelity DNA polymerase and sequenced. Vertis Biotechnologie AG removed the adapters from the sequences and did a preliminary quality control of the data. RNA-Seq data was analyzed following the workflow described in^47^. In summary, the RNA-Seq data quality was confirmed using fastQC program. We mapped the reads against *E. coli* genome (NC_000913 downloaded from NCBI genome database) using Bowtie2 program^48^ and obtained more than 90% of aligned reads. The mapping files were sorted by genomic position using the Samtools^49^ and the quantification of the transcripts expression was done using the Artemis software^50^. The differential expression analysis was done with the R package edgeR^51^. We considered all transcripts with a False Discovery Rate (FDR) correction of the p-value lower than 0.05 as significant and we further filtered our results using the expression values (LogCPM) higher than 3 and a fold-change between two samples higher than two. The functional annotation was performed with DAVID functional annotation tool^52^.

### cDNA synthesis and qPCR

We synthesized cDNA for quantitative RT-PCR using the SensiFAST™ cDNA Synthesis Kit (Bioline). The RT-PCR was performed with a Corbett Rotor Gene RG 3000 real-time PCR system and SensiFAST SYBR No-ROX Kit (Bioline). Primers for qPCR are listed in Table S12 and the parameters for qPCR were: 95 °C for 2 min, 40 cycles of 95 °C for 10 sec, 60 °C for 15 sec, 72 °C for 20 sec. Each run had a negative control (without cDNA) and a melting curve was obtained from a first step starting from 60 to 95 °C, to control specificities of quantitative PCR reaction for each primer pair. A standard curve with several cDNA dilutions (1:5, 1:10, 1:20, 1:50 and 1:75) was used to determine the efficiency of amplifications. Relative copy number was calculated with the ΔΔCt method and using *ihfB* (for stress response transcripts), *rrfH* (for comparing exponential with stationary phase in the double mutant) or 23S (for all other comparisons) as the reference gene. qPCR was performed in triplicate with, at least, three templates of RNA extracted from independent cultures.

## Supplementary information

Supplementary data

Supplementary Tables

## Data Availability

The data discussed in this publication have been deposited in NCBI’s Gene Expression Omnibus^53^ and are accessible through GEO Series accession number GSE60107 and GSE117635. Other supporting data are included as additional files.

## References

[1] Ishihama A (1997). Adaptation of gene expression in stationary phase bacteria. Curr Opin Genet Dev.

[2] Kolter R, Siegele DA, Tormo A (1993). The stationary phase of the bacterial life cycle. Annu Rev Microbiol.

[3] Navarro Llorens JM, Tormo A, Martinez-Garcia E (2010). Stationary phase in gram-negative bacteria. FEMS Microbiol Rev.

[4] Hengge-Aronis R (1999). Interplay of global regulators and cell physiology in the general stress response of *Escherichia coli*. Curr Opin Microbiol.

[5] Geissen R, Steuten B, Polen T, Wagner R (2010). *E. coli* 6S RNA: a universal transcriptional regulator within the centre of growth adaptation. RNA Biol.

[6] Barnett TC, Bugrysheva JV, Scott JR (2007). Role of mRNA stability in growth phase regulation of gene expression in the group A streptococcus. J Bacteriol.

[7] Nilsson G, Belasco JG, Cohen SN, von Gabain A (1984). Growth-rate dependent regulation of mRNA stability in *Escherichia coli*. Nature.

[8] Cheng ZF, Zuo Y, Li Z, Rudd KE, Deutscher MP (1998). The *vacB* gene required for virulence in *Shigella flexneri* and *Escherichia coli* encodes the exoribonuclease RNase R. J Biol Chem.

[9] Donovan WP, Kushner SR (1986). Polynucleotide phosphorylase and ribonuclease II are required for cell viability and mRNA turnover in *Escherichia coli* K-12. Proc Natl Acad Sci USA.

[10] Andrade JM, Pobre V, Silva IJ, Domingues S, Arraiano CM (2009). The role of 3′-5′ exoribonucleases in RNA degradation. Prog Mol Biol Transl Sci.

[11] Dos Santos RF (2018). Major 3′-5′ Exoribonucleases in the Metabolism of Coding and Non-coding RNA. Prog Mol Biol Transl Sci.

[12] Reis FP, Pobre V, Silva IJ, Malecki M, Arraiano CM (2013). The RNase II/RNB family of exoribonucleases: putting the ‘Dis’ in disease. Wiley Interdiscip Rev RNA.

[13] Frazão C (2006). Unravelling the dynamics of RNA degradation by ribonuclease II and its RNA-bound complex. Nature.

[14] Awano N (2010). *Escherichia coli* RNase R has dual activities, helicase and RNase. J Bacteriol.

[15] Hossain ST, Malhotra A, Deutscher MP (2016). How RNase R Degrades Structured RNA: Role of The Helicase Activity and The s1 Domain. J Biol Chem.

[16] Spickler C, Mackie A (2000). Action of RNases II and Polynucleotide Phosphorylase against RNAs containing stem-loops of defined structure. J. Bacteriol..

[17] Hajnsdorf E, Steier O, Coscoy L, Teysset L, Régnier P (1994). Roles of RNase E, RNase II and PNPase in the degradation of the *rpsO* transcripts of *Escherichia coli*: stabilizing function of RNase II and evidence for efficient degradation in an *ams pnp rnb* mutant. EMBO J.

[18] Marujo PE (2000). RNase II removes the oligo(A) tails that destabilize the *rpsO* mRNA of *Escherichia coli*. RNA.

[19] Mohanty, B. K. & Kushner, S. R. Polynucleotide phosphorylase, RNase II and RNase E play different roles in the *in vivo* modulation of polyadenylation in *Escherichia coli*. *Mol Microbiol* **36**, 982–994, mmi1921 (2000). 10.1046/j.1365-2958.2000.01921.x 10844684

[20] Pepe CM, Maslesa-Galic S, Simons RW (1994). Decay of the IS10 antisense RNA by 3′ exoribonucleases: evidence that RNase II stabilizes RNA-OUT against PNPase attack. Mol Microbiol.

[21] Liang W, Malhotra A, Deutscher MP (2011). Acetylation regulates the stability of a bacterial protein: growth stage-dependent modification of RNase R. Mol Cell.

[22] Song L, Wang G, Malhotra A, Deutscher MP, Liang W (2016). Reversible acetylation on Lys501 regulates the activity of RNase II. Nucleic Acids Res.

[23] Pobre V, Arraiano CM (2015). Next generation sequencing analysis reveals that the ribonucleases RNase II, RNase R and PNPase affect bacterial motility and biofilm formation in *E. coli*. BMC Genomics.

[24] Malecki M, Bárria C, Arraiano CM (2014). Characterization of the RNase R association with ribosomes. BMC Microbiol.

[25] Liang W, Deutscher MP (2013). Ribosomes regulate the stability and action of the exoribonuclease RNase R. J Biol Chem.

[26] Grunberg-Manago M, Oritz PJ, Ochoa S (1955). Enzymatic synthesis of nucleic acidlike polynucleotides. Science.

[27] Godefroy T (1970). Kinetics of polymerization and phosphorolysis reactions of *Escherichia coli* polynucleotide phosphorylase. Evidence for multiple binding of polynucleotide in phosphorolysis. Eur J Biochem.

[28] Mohanty BK, Kushner SR (2000). Polynucleotide phosphorylase functions both as a 3′ right-arrow 5′ exonuclease and a poly(A) polymerase in *Escherichia coli*. Proc Natl Acad Sci USA.

[29] Miczak A, Kaberdin VR, Wei CL, Lin-Chao S (1996). Proteins associated with RNase E in a multicomponent ribonucleolytic complex. Proc Natl Acad Sci USA.

[30] Py B, Causton H, Mudd EA, Higgins CF (1994). A protein complex mediating mRNA degradation in *Escherichia coli*. Mol Microbiol.

[31] Py B, Higgins CF, Krisch HM, Carpousis AJ (1996). A DEAD-box RNA helicase in the *Escherichia coli* RNA degradosome. Nature.

[32] Vanzo NF (1998). Ribonuclease E organizes the protein interactions in the *Escherichia coli* RNA degradosome. Genes Dev.

[33] Andrade JM, Pobre V, Matos AM, Arraiano CM (2012). The crucial role of PNPase in the degradation of small RNAs that are not associated with Hfq. RNA.

[34] Andrade JM, Pobre V, Arraiano CM, Small RNA (2013). modules confer different stabilities and interact differently with multiple targets. PLoS One.

[35] Saramago M (2014). The role of RNases in the regulation of small RNAs. Curr Opin Microbiol.

[36] Andrade JM, Arraiano CM (2008). PNPase is a key player in the regulation of small RNAs that control the expression of outer membrane proteins. RNA.

[37] Bandyra KJ, Sinha D, Syrjanen J, Luisi BF, De Lay NR (2016). The ribonuclease polynucleotide phosphorylase can interact with small regulatory RNAs in both protective and degradative modes. RNA.

[38] De Lay N, Gottesman S (2011). Role of polynucleotide phosphorylase in sRNA function in *Escherichia coli*. RNA.

[39] Cameron TA, De Lay NR (2016). The Phosphorolytic Exoribonucleases Polynucleotide Phosphorylase and RNase PH Stabilize sRNAs and Facilitate Regulation of Their mRNA Targets. J Bacteriol.

[40] Cardenas PP (2009). *Bacillus subtilis* polynucleotide phosphorylase 3′-to-5′ DNase activity is involved in DNA repair. Nucleic Acids Res.

[41] Danchin A (1997). Comparison between the Escherichia coli and Bacillus subtilis genomes suggests that a major function of polynucleotide phosphorylase is to synthesize CDP. DNA Res.

[42] de Bruijn Frans J. (2016). Stress and Environmental Regulation of Gene Expression and Adaptation in Bacteria.

[43] Cairrão F, Cruz A, Mori H, Arraiano CM (2003). Cold shock induction of RNase R and its role in the maturation of the quality control mediator SsrA/tmRNA. Mol Microbiol.

[44] Chen HW (2006). Mammalian polynucleotide phosphorylase is an intermembrane space RNase that maintains mitochondrial homeostasis. Mol Cell Biol.

[45] Del Favero M (2008). Regulation of *Escherichia coli* polynucleotide phosphorylase by ATP. J Biol Chem.

[46] Simonte FM, Dotsch A, Galego L, Arraiano C, Gescher J (2017). Investigation on the anaerobic propionate degradation by Escherichia coli K12. Mol Microbiol.

[47] Pobre V, Arraiano CM (2018). Characterizing the Role of Exoribonucleases in the Control of Microbial Gene Expression: Differential RNA-Seq. Methods Enzymol.

[48] Langmead B, Salzberg SL (2012). Fast gapped-read alignment with Bowtie 2. Nat Methods.

[49] Li H (2009). The Sequence Alignment/Map format and SAMtools. Bioinformatics.

[50] Carver T, Harris SR, Berriman M, Parkhill J, McQuillan JA (2012). Artemis: an integrated platform for visualization and analysis of high-throughput sequence-based experimental data. Bioinformatics.

[51] McCarthy DJ, Chen Y, Smyth GK (2012). Differential expression analysis of multifactor RNA-Seq experiments with respect to biological variation. Nucleic Acids Res.

[52] Huang da W, Sherman BT, Lempicki RA (2009). Systematic and integrative analysis of large gene lists using DAVID bioinformatics resources. Nat Protoc.

[53] Edgar R, Domrachev M, Lash AE (2002). Gene Expression Omnibus: NCBI gene expression and hybridization array data repository. Nucleic Acids Res.

