# Supplementary material for: Defining the impact of exoribonucleases in the shift between exponential and stationary phases
Source: Sci Rep. 2019 Nov 7;9:16271. doi: 10.1038/s41598-019-52453-6 (PMC6838162; doi:10.1038/s41598-019-52453-6)
Supplement: Supplementary file 1 — Supplementary data [file 41598_2019_52453_MOESM1_ESM.docx]

**Defining the impact of exoribonucleases**

**in the shift between exponential and stationary phases**

**Supplementary data**

Vânia Pobre^1*^, Susana Barahona^1^, Tatiane Dobrzanski^2^, Maria Berenice Reynaud Steffens^2^, Cecília M. Arraiano^1*^

**This supplement contains Figures S1, Figure S2, Figure S3, Table S11, Table S12 and Supplementary References**

**Fig S1 – Global changes between WT and the exoribonuclease single mutants in Stationary phase**


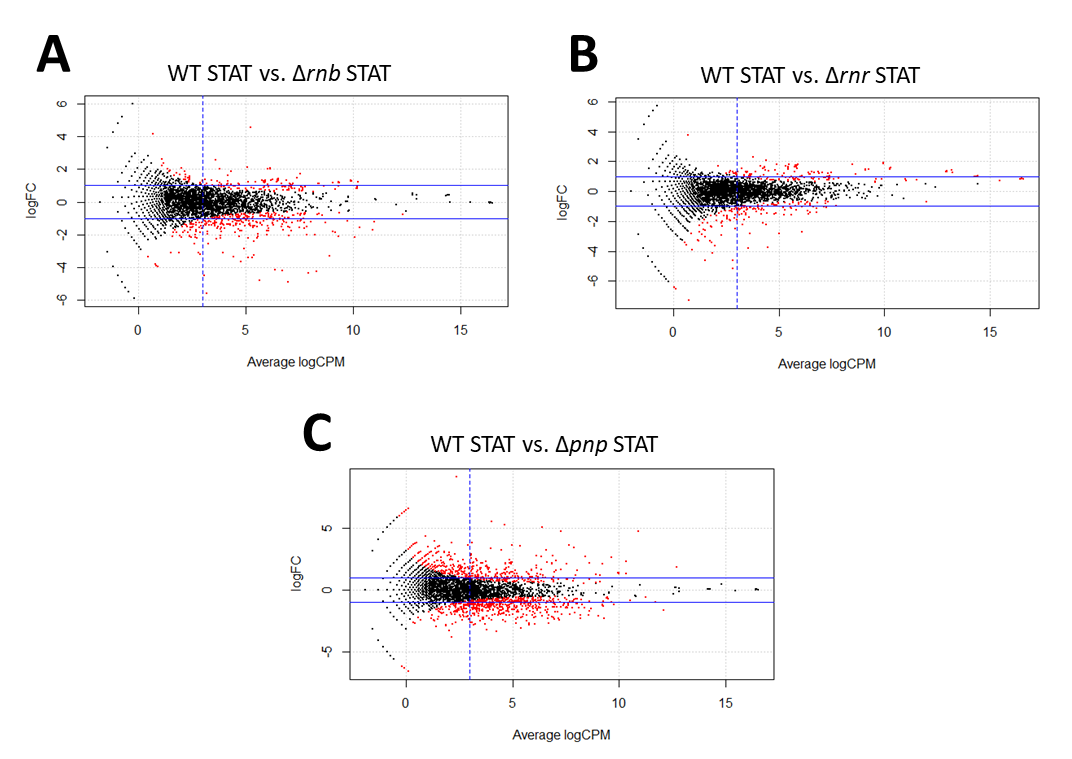


**A)** MA scatterplot comparing wild-type (WT) with Δ*rnb* mutant in stationary phase. **B)** MA scatterplot comparing wild-type (WT) with Δ*rnr* mutant in stationary phase. **C)** MA scatterplot comparing wild-type (WT) with Δ*pnp* mutant in stationary phase. LogFC is the log2 of the fold change for each transcript, average LogCPM is the relative expression value for each transcript. Transcripts in red were considered significantly differentially expressed (FDR<0.5), the two horizontal blue lines correspond to a fold-change of 2 and the vertical blue line correspond to the LogCPM of 3. These lines represent the filtration steps done to obtain the final list of differentially expressed transcripts.

**Fig. S2 – Pathways affected in the exoribonuclease mutants when compared to the WT in stationary phase**


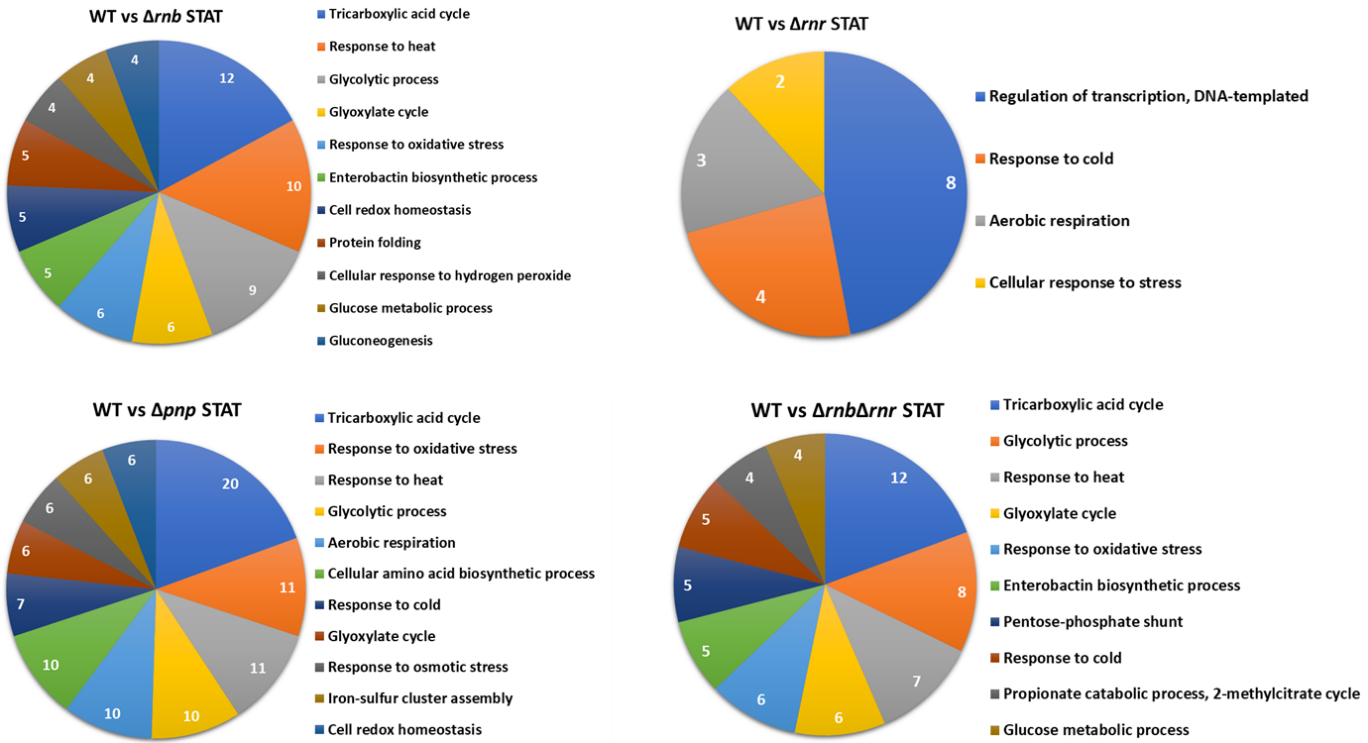


Functional annotation of the differentially expressed transcripts between the WT and the different exoribonuclease mutants in stationary phase. Transcripts were grouped into different functional categories but only the Gene Ontology category of biological process is represented.

**Fig. S3 – Growth curves for the strains analysed in this work**


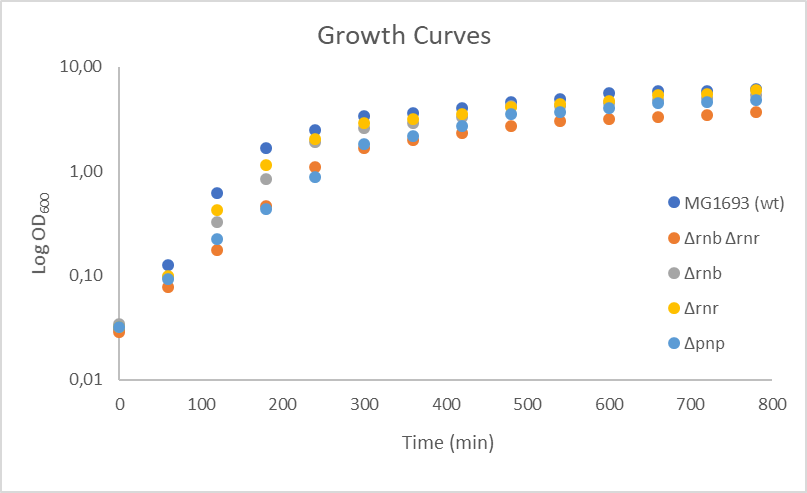


*E. coli* K-12 strain MG1693 and its isogenic mutants were grown at 37°C, with shaking at 200 rpm in Luria-Bertani (LB) medium supplemented with thymine (50 μg ml^−1^). Antibiotics were present at the following concentrations: kanamycin, 50 μg ml^−1^ (Δ*rnr*); tetracycline, 20 μg ml^−1^ (Δ*rnb*); streptomycin/spectinomycin 20 μg ml^−1^ (Δ*pnp*); kanamycin, 50 μg ml^−1^/ tetracycline, 20 μg ml^−1^ (Δ*rnb* Δ*rnr*).

**Table S11 – Bacterial strains used in this study**

| **Strain** | **Relevant genotype** | **Reference** |
| --- | --- | --- |
| MG1693 | thyA715 | [^1^](#_ENREF_1) |
| CMA201 | thyA715 Δ*rnb* | [^2^](#_ENREF_2) |
| HM104 | thyA715 Δ*rnr* | [^2^](#_ENREF_2) |
| SK10019 | thyA715 Δ*pnp* | [^3^](#_ENREF_3) |
| HM103 | thyA715 Δ*rnb*Δ*rnr* | [^4^](#_ENREF_4) |

**Table S12 – Primers used in this study**

| **Primer** | **Sequence (5’-3’)** |
| --- | --- |
| 23S-FW | TAAGCGTCGCTGCCG |
| 23S-Rev | AAAGAAAGCGTAATAGCTCACTGGTC |
| aceA-FW | GAATTTGCCGCCGCGAAAGATG |
| aceA-Rev | AACCTGTACCCACTTCCTGCTG |
| acnA-FW | TATCGGCCATGATCGCAAAGGC |
| acnA-Rev | CTACCGCACGGGCAATTTCTTG |
| clpB-FW | TCGCCCGATTGGTTCATTCCTG |
| clpB-Rev | AGTTCGCCAGCGCCTTACAAAG |
| cyoB-FW | ATCTGGTGGCTGGCGATTGTTG |
| cyoB-Rev | TAATCCACGTCCTCGTCGAAGC |
| dmsA-FW | CCGATCCAGACCAGCATTT |
| dmsA-Rev | ATCGGCACATCCAGCTTATC |
| dnaB-FW | GCACTGGCGAAAGAACTGAACG |
| dnaB-Rev | TTGTCGGCACGTTGTTCCAGAG |
| dnaK-FW | GAAGAAGCAGGCGACAAACTGC |
| dnaK-Rev | AGCAGTTTCCAGTGCAGTCAGC |
| fecC-FW | TTCGATCAGCGCAACGTACTGC |
| fecC-Rev | TACATCTGCCAGCAGCATCAGC |
| fhuB-FW | GGGCGTTGCTCGAGGATTTAATG |
| fhuB-Rev | CAAACAGCGCCGCCATAATTCG |
| frdA-FW | ACCGACCTGCTCTACACCATTG |
| frdA-Rev | TTGCGGAGTGCGCCATACATTC |
| fre-FW | ACGTCCGTTCTCAATGGCTTCG |
| fre-Rev | TCAGAAGCGCCAATATGCAGCTC |
| gltA-FW | GCCGGTAACTTCCTGAATATGA |
| gltA-Rev | CAGCGTGCAGGATCAGAATA |
| groL-FW | TCCGCTGCGTCAGATCGTATTG |
| groL-Rev | TCGCCGCCTTTAACGGTGTTAG |
| holA-FW | ACTGGAGCGTTTATCGCTGCTC |
| holA-Rev | TCATTCACCGCCTGTTCAACGC |
| htpG-FW | AACCAGGAAGCGATCGCCAAAC |
| htpG-Rev | TGCGCAGAAGAATCGGTATGGG |
| ibpA-FW | TCGCTGAACGCAACTTTGAACG |
| ibpA-Rev | TTTACCAGGTTAGCACCACGAAC |
| icd-FW | TTCGCTTCCCGGAACATTGTGG |
| icd-Rev | AGACGTTTGGTGCCTTCTTCCG |
| ihfB-FW | AGCATATGGCCTCGACTCTTGC |
| ihfB-Rev | GCGCGGTAGTGCAAAGAGAAAC |
| katE-FW | ACGCGCGCAAGTTTAAAGCAAC |
| katE-Rev | AGCGCTGTCAGCTTCCACAATC |
| katG-FW | ATAGTGCTGGCTGGTGTGGTTG |
| katG-Rev | GGCGCAAACGGTACATGAATGC |
| lpxK-FW | TTGCGGTTTCTCCCGTTCGTTC |
| lpxK-Rev | ATCTGCACATCAGGGTGTTGCG |
| mukB-FW | TGAACAGCACGAGCAGCATCAG |
| mukB-Rev | AGCGCTTCCGAGAAGGTCAAAC |
| napA-FW | CCTTCGATGTGATGGAAGAGAA |
| napA-Rev | CGGCATAACCTTCCCAGATAG |
| rrfH-FW | CATGCCGAACTCAGAAGTGAA |
| rrfH-Rev | CCTGGCAGTTCCCTACTCT |
| rsxC-FW | CTGGCGGACTCTAACGATATTT |
| rsxC-Rev | CCCGGTCAGAATGTAGGTTAAT |
| sdhC-FW | ATCACGGCGATAGCGTCCATTC |
| sdhC-Rev | AGGATGCCCACTGCAACAAAGG |
| sucD-FW | CACATTCACAAACCGGGTAAAG |
| sucD-Rev | CGACTGACCGAAACCGTAAT |
| waaC-FW | GGCACTGGATAGACCCAATATC |
| waaC-Rev | TGGAGCCCTACATACCATCT |
| waaY-FW | GGTGAGGTTCGAATTATCGATCT |
| waaY-Rev | CCGTAATGACGCTCTAAGTCAA |

**Supplementary References**

1 Arraiano, C. M., Yancey, S. D. & Kushner, S. R. Stabilization of discrete mRNA breakdown products in *ams pnp rnb* multiple mutants of *Escherichia coli* K-12. *J. Bacteriol.* **170**, 4625-4633 (1988).

2 Andrade, J. M., Cairrão, F. & Arraiano, C. M. RNase R affects gene expression in stationary phase: regulation of *ompA*. *Mol. Microbiol.* **60**, 219-228, doi:MMI5092 [pii]10.1111/j.1365-2958.2006.05092.x (2006).

3 Mohanty, B. K. & Kushner, S. R. Genomic analysis in *Escherichia coli* demonstrates differential roles for polynucleotide phosphorylase and RNase II in mRNA abundance and decay. *Mol. Microbiol.* **50**, 645-658, doi:3724 [pii] (2003).

4 Viegas, S. C., Schmidt, D., Kasche, V., Arraiano, C. M. & Ignatova, Z. Effect of the increased stability of the penicillin amidase mRNA on the protein expression levels. *FEBS Lett* **579**, 5069-5073, doi:S0014-5793(05)00997-X [pii]10.1016/j.febslet.2005.08.018 (2005).
